# SHIVA - a web application for drug resistance and tropism testing in HIV

**DOI:** 10.1186/s12859-016-1179-2

**Published:** 2016-08-22

**Authors:** Mona Riemenschneider, Thomas Hummel, Dominik Heider

**Affiliations:** 1 Department of Bioinformatics, Straubing Center of Science, Petersgasse 18, Straubing, 94315 Germany; 2 University of Applied Science Weihenstephan-Triesdorf, Am Hofgarten 4, Freising, 85354 Germany; 3 Wissenschaftszentrum Weihenstephan, Technische Universität München, Alte Akademie 8, Freising, 85354 Germany

**Keywords:** Infectious diseases, Machine learning, Retrovirus, HIV therapy

## Abstract

**Background:**

Drug resistance testing is mandatory in antiretroviral therapy in human immunodeficiency virus (HIV) infected patients for successful treatment. The emergence of resistances against antiretroviral agents remains the major obstacle in inhibition of viral replication and thus to control infection. Due to the high mutation rate the virus is able to adapt rapidly under drug pressure leading to the evolution of resistant variants and finally to therapy failure.

**Results:**

We developed a web service for drug resistance prediction of commonly used drugs in antiretroviral therapy, i.e., protease inhibitors (PIs), reverse transcriptase inhibitors (NRTIs and NNRTIs), and integrase inhibitors (INIs), but also for the novel drug class of maturation inhibitors. Furthermore, co-receptor tropism (CCR5 or CXCR4) can be predicted as well, which is essential for treatment with entry inhibitors, such as Maraviroc. Currently, SHIVA provides 24 prediction models for several drug classes. SHIVA can be used with single RNA/DNA or amino acid sequences, but also with large amounts of next-generation sequencing data and allows prediction of a user specified selection of drugs simultaneously. Prediction results are provided as clinical reports which are sent via email to the user.

**Conclusions:**

SHIVA represents a novel high performing alternative for hitherto developed drug resistance testing approaches able to process data derived from next-generation sequencing technologies. SHIVA is publicly available via a user-friendly web interface.

## Background

Successful antiretroviral therapy (ART) in HIV infected patients strongly depends on the effective suppression of viral replication. Although a broad range of antiretroviral drugs have been approved by the FDA, long-lasting reduction of virus load is still challenging today. The high mutation rate of the virus under drug pressure remains the major concern in ART. In general, three drugs of different drug classes, e.g., protease inhibitors (PIs), reverse transcriptase inhibitors (NRTIs and NNRTIs), or entry inhibitors, are administered to the patient in a combined therapy. However, as an adaption process the virus is able to evade ART regimes finally leading to therapy failure. Therefore, predicting drug resistances or co-receptor usage, respectively, is an essential procedure in the efficient suppression of virus load and though in prolonging a patient’s life. Overall, six drug classes are at present available that target diverse stages within the replication cycle of HI viruses. Nucleotide reverse transcriptase inhibitors (NRTIs) and non-nucleoside reverse transcriptase inhibitors (NNRTIs) are able to block the activity of the reverse transcriptase therewith disrupting the building process of viral DNA, which finally leads to the inhibition of the virus’ life cycle. Protease inhibitors (PIs) suppress the function of the protease enzyme, which is responsible for cutting the gag polypeptide into functional proteins. Integrase inhibitors (INIs) prevent the insertion of viral DNA into the host cell by inhibiting the viral integrase. Another prevention of virus replication can be achieved by inhibiting the entry of viruses into host cells. In general, the HI virus binds to the CD4 receptor of CD4 expressing cells, and additionally, to one of two co-receptors, either CXCR4 or CCR5. The engagement is crucial to activate the fusion of virus and host cell [1]. Entry-inhibitors, such as Maraviroc [2], are able to block the co-receptor binding to CCR5 and therefore inhibit virus replication. However, before administering entry-inhibitors to the patient it is necessary to predict co-receptor usage of the viral population, as entry-inhibitors are only effective against CCR5-tropic viruses. A rather new antiretroviral drug, Bevirimat (BVM) [3], has been evaluated for HIV therapy, albeit not yet approved by the FDA for clinical application. BVM is a maturation inhibitor that inhibits the maturation of HIV particles to infectious virions by preventing the final cleavage of the precursor protein p25 to p24 and p2. However, the emergence of drug resistances is the major impediment in effective ART. Thus, sophisticated computational algorithms have been developed to predict drug resistances on viral sequences. For example, geno2pheno [4, 5], HIVdb [6], and WebPSSM [7] are the most popular tools in resistance testing and co-receptor tropism prediction. However, there is still the great need to further improve prediction algorithms for HIV drug resistance and tropism prediction. For example, Dybowski et al. [8] have shown the potential of random forest [9] models on HIV tropism prediction, outperforming prediction performances of geno2pheno and other methods.

We developed a web service for HIV drug resistance prediction incorporating models for resistance testing of PIs, NNRTIs, NRTIs, INIs, BVM, as well as co-receptor tropism prediction. The algorithm design is able to handle up to several million sequences therefore allowing queries with next-generation sequencing (NGS) data [10]. Prediction results are provided as clinical reports presenting the results in a comprehensible and clearly presented way and sent via email to the user. Additionally, a data file containing resistance information for each sequence is generated thus providing also detailed information for all sequences within a patient. The graphical user interface makes the application handy for researchers as well as for clinicians. The application SHIVA is available as a web interface with public access at http://shiva.heiderlab.de.

## Implementation

The web application is based on the JAVA framework Vaadin (https://vaadin.com) and can be accessed via a web browser. Drug resistance models that are incorporated into and provided by SHIVA were implemented in R (http://www.r-project.org), thus requiring communication and data transfer between JAVA and R, which is handled using the Rserve package. The analytical report generation is conducted using the JasperReports library. For processing biological data, e.g., for translating RNA sequences into protein sequences, the BioJava library [11] is used.

The models that were incorporated into SHIVA so far provide predictions for six antiretroviral drug classes comprising the following 23 drugs: PIs (Amprenavir, Atazanavir, Indinavir, Lopinavir, Ritonavir, Saquinavir, Nelfinavir, Darunavir), NRTIs (Lamivudine, Abacavir, Zidovudine, Didanosine, Tenofovir, Stavudine, Emtricitabine), NNRTIs (Efavirenz, Nevirapine, Delavirdine, Rilpivirine), INIs (Dolutegravir, Elvitegravir, Raltegravir), and the maturation inhibitor Bevirimat. Furthermore, a prediction model for co-receptor tropism prediction has been incorporated. For most of the drugs (except Darunavir, Emtricitabine, and the INIs) resistance prediction is performed by random forests models developed in our group recently [12, 13]. These models are based on independent binary classification for each drug, predicting sequences as resistant or susceptible using a drug-specific cutoff (see [12, 13]). The random forests have been trained with 500 trees and have been evaluated with a leave-one-out cross-validation scheme.

Random forests [9] are ensemble learning methods for classification and regression, consisting of multiple decision trees, which predict the drug resistance for a given sequence independently of each other. The predictions are then combined via majority voting to get a final decision, i.e., resistant or susceptible.

For feature representation, sequences were translated to numerical values using the hydrophobicity scores according to Kyte and Doolittle [14]. Due to varieties in sequence lengths, an interpolation of input sequences was conducted with Interpol [15] to vectors of lengths 99 for PIs and 240 for NNRTIs and NRTIs, respectively. Feature length of p2 sequences for BVM prediction was interpolated to 20. For the prediction of co-receptor usage, we incorporated TCUP 2.0 [16] into SHIVA, which is based on a stacking approach of two random forest models. One random forest model is trained on the numerical representation of the V3 loop using hydrophobicity values, the other one is trained on the electrostatic potential of the V3 loop. Independent predictions are then combined via stacking to get the final prediction result for each sequence. Besides using data-driven approaches for drug resistance, we also employed knowledge-based approaches for the INIs, Emtricitabine, and Darunavir based on the 2015 edition of the IAS-USA drug resistance mutations list [17].

## Results and discussion

**Fig. 1:**
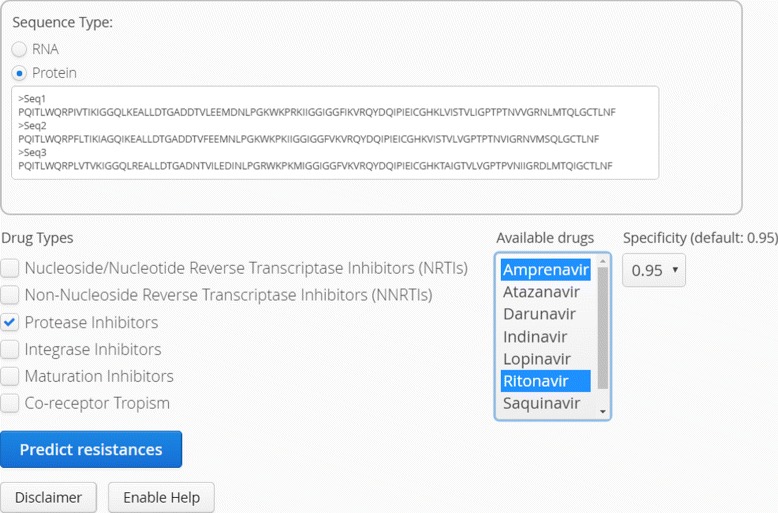
Web interface of SHIVA: Sequences can be pasted directly into the input form or uploaded as FASTA files. Drugs can be selected via checkboxes. The specificity cut-off can be selected as 0.9, 0.95, or 0.98

**Fig. 2:**
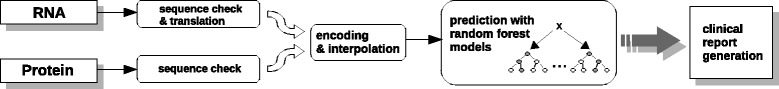
Workflow of drug resistance prediction: First, protein as well as RNA/DNA sequences are quality controlled. The latter ones are then translated into protein sequences. The second steps includes descriptor encoding and interpolation. Next, the drug resistance/tropism is predicted on a per sequences level. Finally, a clinical report is generated

**Fig. 3:**
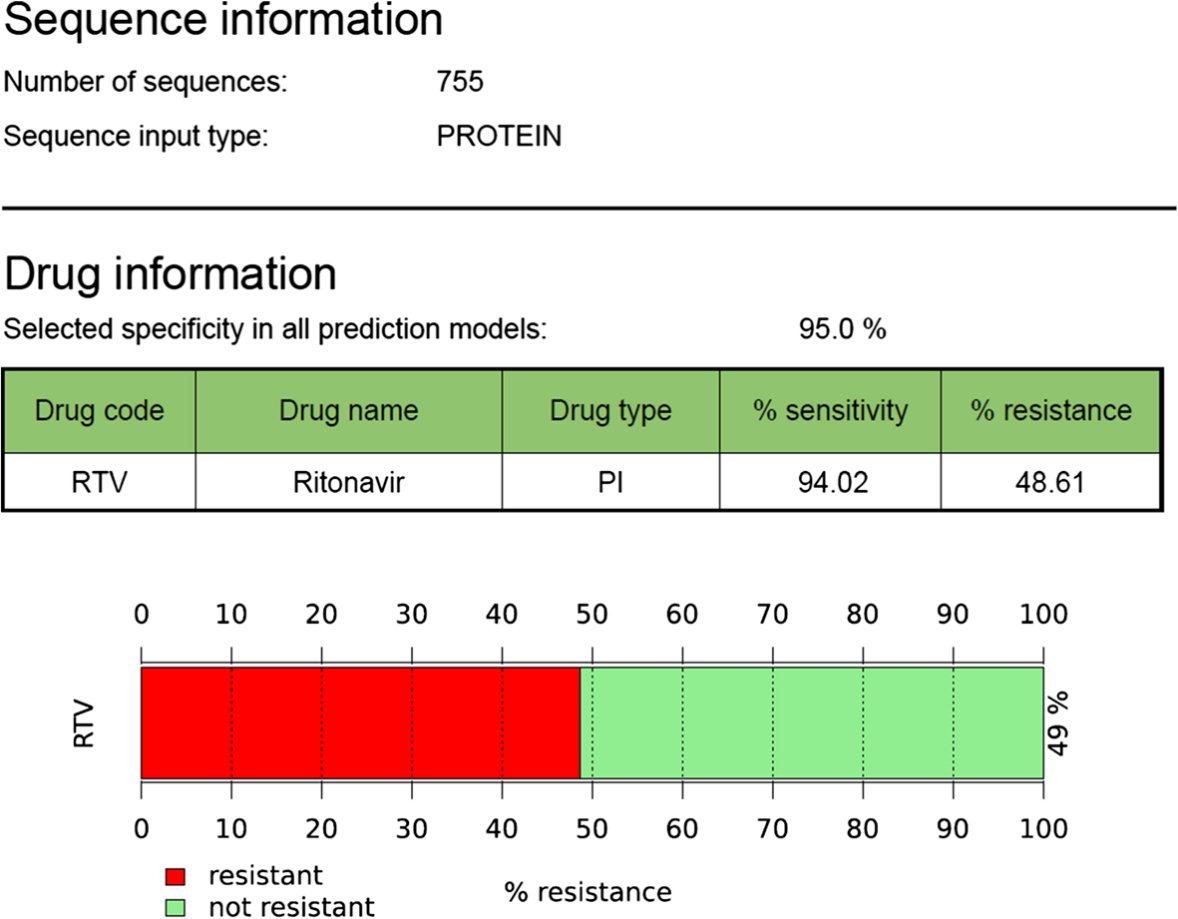
Clinical report: Drug resistance predictions are listed in tabular form and are graphically represented demonstrating the fraction of resistant and susceptible, i.e., non-resistant sequences. Here, prediction results are shown for an example dataset to test resistance against Ritonavir (RTV). 48.61 % of the input protease sequences derived from Sanger sequencing have been predicted to be resistant with a sensitivity of 94.02 %

The web application SHIVA (Fig. 1) has been developed to provide researchers and clinicians an easy-to-use interface to our recently developed drug resistance and tropism prediction models. At the moment, there are drug resistance models for 23 drugs (PIs, NRTIs, NNRTIs, INIs, and BVM) available. Moreover, it can be used to predict co-receptor tropism based on V3 loop sequence data. Data input must be provided as RNA/DNA or protein sequences in FASTA format. RNA/DNA sequences are translated into amino acid representations. Additionally, sequences are checked for input format and illegal characters. To provide the appropriate input format for the prediction models, the protein sequences are encoded with hydrophobicity values. For co-receptor usage prediction, the electrostatic potentials of the V3 loop sequences are calculated by TCUP 2.0 [16]. For each prediction query the false positive rate can be controlled by setting a cut-off for the specificity. The default value is 0.95 for drug resistance predictions and 0.98 for co-receptor determination. Figure 2 demonstrates the resistance testing workflow. The results of the predictions are provided as clinical reports in the web browser and can be optionally sent via email to the user. These reports contain the following information:

1. user information, such as user ID, a patient’s ID (if provided), and date of query,
2. information about input data, i.e., number of sequences, the type of sequence (RNA/DNA or protein), and the list of sequences (only when a small set of sequences has been queried),
3. prediction results, i.e., the prediction results for queried drugs, the selected specificity cut-off and a visualization for easy interpretation of prediction results (see Fig. 3).

The clinical report shows the fraction of resistant sequences for a given patient, which can be used to detect resistant minority variants. Moreover, the report gives information about the drug model used and its general sensitivity for the selected specificity cut-off. For instance, as shown in Fig. 3, the sensitivity of the model predicting Ritonavir resistance is 94.02 *%* for the selected specificity of 95.0 *%*, and the fraction of resistant sequences for the given patient is 48.61 *%*.

A comparison between the prediction servers SHIVA, geno2pheno (resistance and coreceptor), HIVdb, and WebPSSM can be found in Table 1. We compared the different drug classes that can be predicted, the maximum number of sequences that can be uploaded, NGS compatability, run time (averaged over 10 runs), whether the server provides a clinical report, and whether the server provides detailed access to the predicted data. In contrast to geno2pheno, HIVdb, and WebPSSM, SHIVA is able to predict NGS data directly. In our recent publications, we already demonstrated the high accuracy of our drug resistance and tropism prediction models compared to other state-of-the-art models, e.g., geno2pheno [4, 5] or HIVdb [6]. Thus, we restricted the comparison of the web-services to features of usability and applicability.

**Table 1:** Comparison between different prediction servers

Server	PIs	NRTIs	NNRTIs	INIs	Mat. Inh.	Tropism	max. #	NGS	Run	Clinical	Detailed
							sequences	data	time^a^	report	data
										t	access
SHIVA	+	+	+	+	+	+	>100,000	all^b^	6.02 /	+	+
									15.22		
g2p _[*resistance*]_	+	+	+	+	-	-	8	-	24.37	+	-
g2p _[*coreceptor*]_	-	-	-	-	-	+	50^c^	454^c^	3.03	+	-
HIVdb	+	+	+	-	-	+	500	-	2.89	(+)	+
WebPSSM	-	-	-	-	-	+	500	-	7.91	-	+

^a^averaged over 10 runs with 8 protease and 50 V3 sequences, respectively

^b^data needs to be provided in FASTA format

^c^Using geno2pheno _[454]_ it is possible to predict >100,000 sequences, however preprocessing of the data needs to be done offline

geno2pheno _*resistance*_ and geno2pheno _*coreceptor*_ are only able to predict up to 8 and 50 sequences, respectively, while HIVdb and WebPSSM are restricted to 500 sequences. For co-receptor prediction based on NGS data generated with 454 pyrosequencing, geno2pheno_454_ can be used as well, however the preprocessing of the data needs to be done offline. There are also differences in run times for the prediction of 8 protease and 50 V3 sequences, respectively. It turned out that HIVdb is the fastest tool, followed by SHIVA with 2.89 and 6.02 seconds for the prediction of 8 protease sequences, respectively. In contrast, geno2pheno _*resistance*_ needs 24.37 seconds. For the prediction of co-receptor tropism, SHIVA is slower than geno2pheno _*coreceptor*_ and WebPSSM, which is mainly due to the internal 3D-modeling process in TCUP 2.0 [16]. Except WebPSSM, all other servers provide a clinical report that can be used by the clinicans, however, the HIVdb report is not very intuitively and thus only of limited use. One major drawback of geno2pheno compared to the other servers is the lack of detailed data access, which is in particular important for large amounts of data.

## Conclusion

SHIVA represents a novel high performing alternative for hitherto developed drug resistance testing approaches. SHIVA allows the processing of large amounts of data derived from high-throughput technologies [18]. Moreover, SHIVA is platform independent, easy to use and publicly available. In future, additional prediction models that are based on multi-label classification techniques and structural descriptors will be incorporated. Recent studies have demonstrated that such approaches have great potential to further improve drug resistance predictions [19, 20]. Moreover, we will incorporate GPU-based implementations [21] of our models in the near future to speed up the prediction of large data sets.

## Availability and requirements

- Project name: SHIVA
- Project home page: http://shiva.heiderlab.de
- Operating system(s): Platform independent
- Programming language: Java, R
- Other requirements: Javascript
- License: GNU LGPL
- Any restrictions to use by non-academics: no licence needed

## Abbreviations

ART: Antiretroviral therapy
BVM: Bevirimat
DNA: Desoxyribonucleic acid
GPU: Graphics processor unit
HIV: Human immunodeficiency virus
ID: identifier
INI: Integrase inhibitor
NGS: Next-generation sequencing
NRTI: Nucleotide reverse transcriptase inhibitor
NNRTI: Non-nucleoside reverse transcriptase inhibitor
PI: Protease inhibitor
RNA: Ribonucleic acid
RTV: Ritonavir

## References

[1] Deng H, Liu R, Ellmeier W, Choe S, Unutmaz D, Burkhart M, Marzio PD, Marmon S, Sutton RE, Hill CM, Davis CB, Peiper SC, Schall TJ, Littman DR, Landau NR (1996). Identification of a major co-receptor for primary isolates of HIV-1. Nature.

[2] Dorr P, Westby M, Dobbs S, Griffin P, Irvine B, Macartney M, Mori J, Rickett G, Smith-Burchnell C, Napier C, Webster R, Armour D, Price D, Stammen B, Wood A, Perros M (2005). Maraviroc (UK-427,857), a potent, orally bioavailable, and selective small-molecule inhibitor of chemokine receptor CCR5 with broad-spectrum anti-human immunodeficiency virus type 1 activity. Antimicrob Agents Chemother..

[3] Salzwedel K, Martin DE, Sakalian M (2007). Maturation inhibitors: a new therapeutic class targets the virus structure. AIDS Rev.

[4] Beerenwinkel N, Schmidt B, Walter H, Kaiser R, Lengauer T, Hoffmann D, Korn K, Selbig J (2002). Diversity and complexity of HIV-1 drug resistance: A bioinformatics approach to predicting phenotype from genotype. Proc Nat Acad Sci USA.

[5] Lengauer T, Sander O, Sierra S, Thielen A, Kaiser R (2007). Bioinformatics prediction of HIV coreceptor usage. Nat Biotechnol..

[6] Liu TF, Shafer RW (2006). Web resources for HIV type 1 genotypic-resistance test interpretation. Clin Infect Dis..

[7] Jensen MA, Li FS, van ’t Wout AB, Nickle DC, Shriner D, He HX, McLaughlin S, Shankarappa R, Margolick JB, Mullins JI (2003). Improved coreceptor usage prediction and genotypic monitoring of R5-to-X4 transition by motif analysis of human immunodeficiency virus type 1 env V3 loop sequences. J Virol..

[8] Dybowski JN, Heider D, Hoffmann D (2010). Prediction of co-receptor usage of HIV-1 from genotype. PLoS Comput Biol.

[9] Breiman L (2001). Random forests. Mach Learn.

[10] Dybowski JN, Heider D, Hoffmann D (2010). Structure of HIV-1 quasi-species as early indicator for switches of co-receptor tropism. AIDS Res Ther.

[11] Holland RC, Down TA, Pocock M, Prlic A, Huen D, James K, Foisy S, Drager A, Yates A, Heuer M, Schreiber MJ (2008). BioJava: an open-source framework for bioinformatics. Bioinformatics.

[12] Heider D, Verheyen J, Hoffmann D (2011). Machine learning on normalized protein sequences. BMC Res Notes.

[13] Heider D, Verheyen J, Hoffmann D (2010). Predicting Bevirimat resistance of HIV-1 from genotype. BMC Bioinformatics.

[14] Kyte J, Doolittle R (1982). A simple method for displaying the hydropathic character of a protein. J Mol Biol..

[15] Heider D, Hoffmann D (2011). Interpol: An R package for preprocessing of protein sequences. BioData Min.

[16] Heider D, Dybowski JN, Wilms C, Hoffmann D (2014). A simple structure-based model for the prediction of HIV-1 co-receptor tropism. BioData Min.

[17] Wensing AM, Calvez V, Günthard HF, Johnson VA, Paredes R, Pillay D, Shafer RW, Richman DD (2015). 2015 update of the drug resistance mutations in hiv-1. Top Antivir Med.

[18] Ramos RT, Carneiro AR, Baumbach J, Azevedo V, Schneider MP, Silva A (2011). Analysis of quality raw data of second generation sequencers with Quality Assessment Software. BMC Res Notes.

[19] Dybowski JN, Riemenschneider M, Hauke S, Pyka M, Verheyen J, Hoffmann D, Heider D (2011). Improved bevirimat resistance prediction by combination of structural and sequence-based classifiers. BioData Min.

[20] Heider D, Senge R, Cheng W, Hüllermeier E (2013). Multilabel classification for exploiting cross-resistance information in HIV-1 drug resistance prediction. Bioinformatics.

[21] Olejnik M, Steuwer M, Gorlatch S, Heider D (2014). gCUP: rapid GPU-based HIV-1 co-receptor usage prediction for next-generation sequencing. Bioinformatics.

